# Impact of add-back FSH on human and mouse prostate following gonadotropin ablation by GnRH antagonist treatment

**DOI:** 10.1530/EC-21-0639

**Published:** 2022-05-16

**Authors:** Eleftherios E Deiktakis, Eleftheria Ieronymaki, Peter Zarén, Agnes Hagsund, Elin Wirestrand, Johan Malm, Christos Tsatsanis, Ilpo T Huhtaniemi, Aleksander Giwercman, Yvonne Lundberg Giwercman

**Affiliations:** 1 Laboratory of Clinical Chemistry, School of Medicine, University of Crete, Heraklion, Greece; 2 Department of Translational Medicine, Lund University, Malmö, Sweden; 3 Imperial College London, Institute of Reproductive and Developmental Biology, London, UK; 4 Malmö University Hospital, Reproductive Medicine Center, Malmö, Sweden

**Keywords:** castration, degarelix, follicle-stimulating hormone, PSA, prostate

## Abstract

**Objective:**

During androgen ablation in prostate cancer by the standard gonadotropin-releasing hormone (GnRH) agonist treatment, only luteinizing hormone (LH) is permanently suppressed while circulating follicle-stimulating hormone (FSH) rebounds. We explored direct prostatic effects of add-back FSH, after androgen ablation with GnRH antagonist, permanently suppressing both gonadotropins.

**Methods:**

The effects of recombinant human (rFSH) were examined in mice treated with vehicle (controls), GnRH antagonist degarelix (dgx), dgx + rFSH, dgx + flutamide, or dgx + rFSH + flutamide for 4 weeks. Prostates and testes size and expression of prostate-specific and/or androgen-responsive genes were measured. Additionally, 33 young men underwent dgx-treatment. Seventeen were supplemented with rFSH (weeks 1–5), and all with testosterone (weeks 4–5). Testosterone, gondotropins, prostate-specific antigen (PSA), and inhibin B were measured.

**Results:**

In dgx and dgx + flutamide treated mice, prostate weight/body weight was 91% lower than in controls, but 41 and 11%, respectively, was regained by rFSH treatment (*P* = 0.02). The levels of seminal vesicle secretion 6, Pbsn, Nkx3.1, beta-microseminoprotein, and inhibin b were elevated in dgx + rFSH-treated animals compared with only dgx treated (all *P* < 0.05). In men, serum inhibin B rose after dgx treatment but was subsequently suppressed by testosterone. rFSH add-back had no effect on PSA levels.

**Conclusions:**

These data provide novel evidence for the direct effects of FSH on prostate size and gene expression in chemically castrated mice. However, in chemically castrated men, FSH had no effect on PSA production. Whether FSH effects on the prostate in humans also require suppression of the residual adrenal-derived androgens and/or a longer period of rFSH stimulation, remains to be explored.

## Introduction

For decades, gonadotropin-releasing hormone (GnRH) agonists have formed the mainstay hormonal treatment of prostate cancer (1). While they generate suppression of testosterone due to persistent suppression of luteinizing hormone (LH), a potential caveat is the rebound of follicle-stimulating hormone (FSH) after its initial suppression. In contrast, the recently introduced GnRH antagonists provide permanent suppression of both gonadotropins (2), which may offer a therapeutic advantage as suggested by the bourgeoning data on extragonadal targets of FSH action, including the prostate gland (3). This conjecture is strengthened by the finding of FSH receptor expression in normal and malignant human prostate cancer parenchyma and neovasculature (4), as well as in human prostate cancer cell lines (5, 6). Furthermore, studies have demonstrated stimulatory effects of FSH signaling in prostate cancer cell lines (7). Very recently, FSH was found to promote human prostate cancer cell line-derived xenograft (PC-3, DU145) growth in intact and degarelix-suppressed nude mice (8). In humans, a crossover study from agonist (leuprolide) to antagonist (degarelix) demonstrated better prostate cancer control with the latter, with a significant decrease in serum prostate-specific antigen (PSA) concentration (9), which was ascribed to the concomitant FSH suppression.

To further study whether FSH can have direct effects on the prostate, we first used an animal model and subsequently conducted an experiment on young healthy volunteers. In both situations, complete gonadotropin deficiency was induced with GnRH antagonist treatment, and prostatic effects were thereafter addressed with add-back FSH supplementation.

## Materials and methods

### Hormonal treatment of animals

**Figure 1:**
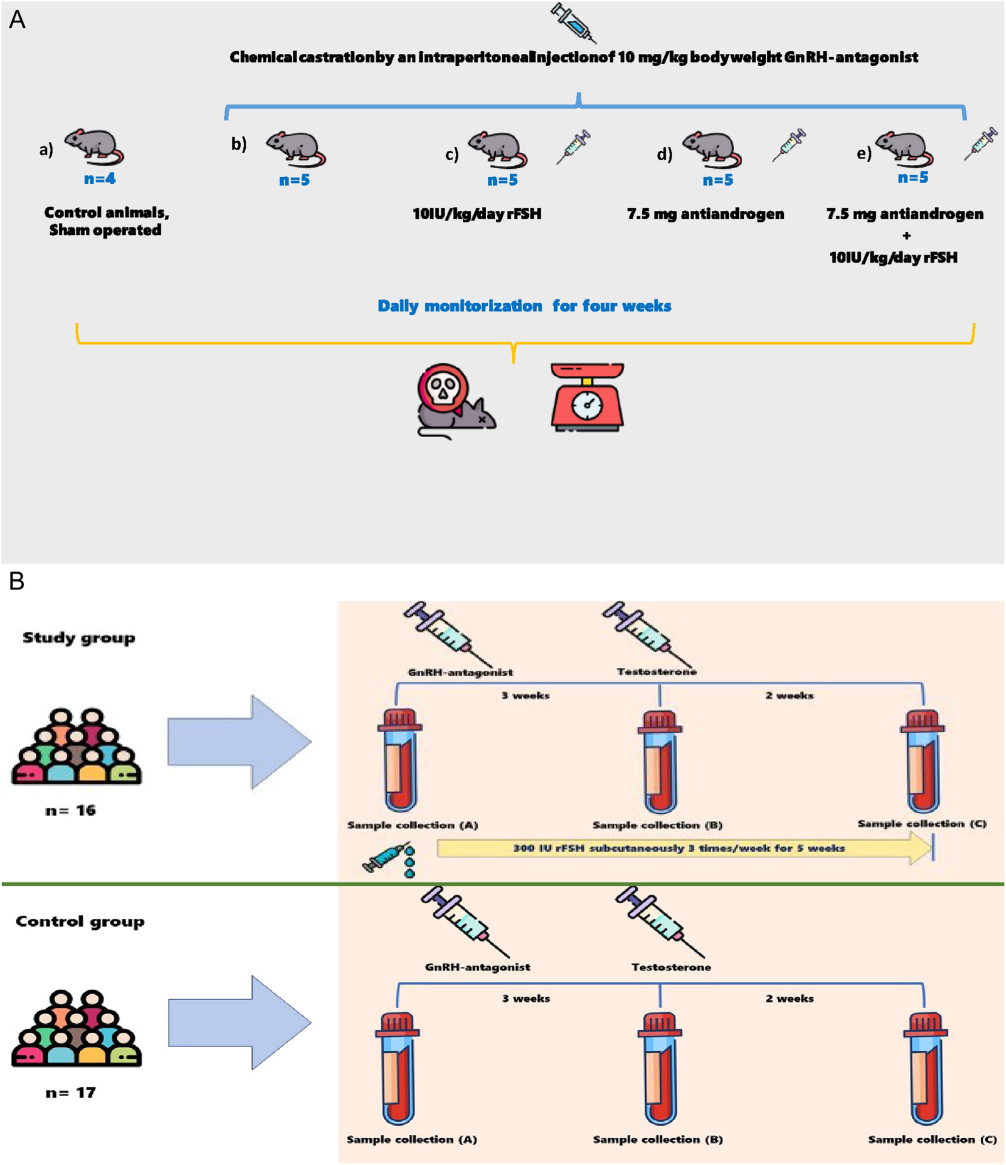
(A) In the animal model, control animals were only sham operated (a). All others (b, c, d, e, f) received an intraperitoneal injection of GnRH antagonist. Animals in group were treated with rFSH (c), whereas one group got antiandrogen only (d) and antiandrogen in combination with rFSH (e). (B) *n* = 33 healthy volunteers received GnRH antagonist at baseline (A). *n* = 16 were randomized to rFSH three times/week for 5 weeks, whereas *n* = 17 served as controls. Blood samples were drawn at baseline (A), 3 weeks after the maximum impact of the GnRH antagonist (B), and at 5 weeks that is 2 weeks after the testosterone injection (C).

Male C57BL/6 mice were weaned 21 days after birth and housed with food and water* ad libitum* at a regular 12-h light cycle and constant temperature. After 6–8 weeks, the mice were stratified into the following treatment groups (Fig. 1A):

- Control animals, sham operated (*n*  = 4).
- Dgx (Firmagon®, Ferring Pharmaceuticals, Saint-Prex, Switzerland; *n* = 5).
- Dgx+rFSH (Gonal-f, Merck Serono S.A. Aubonne, Switzerland; *n* = 5).
- Dgx+antiandrogen (Flutamide, STADA Nordic ApS, Herlev, Denmark; *n* = 5).
- Dgx+antiandrogen+rFSH (*n*  = 5).

Chemical castration was achieved by an intraperitoneal injection of 10 mg/kg body weight dgx. Groups c and e were supplemented with 10 IU/kg/day rFSH using Alzet® osmotic pumps (AgnTho’s AB, Lidingö, Sweden). Animals in groups d and e received 7.5 mg slow-release pellets of the antiandrogen flutamide to inhibit the action of the residual adrenal androgens (10). Flutamide pellets and Alzet pumps were inserted subcutaneously underneath the dorsal skin through a 1-cm incision. The mice were anesthetized prior to the procedures by intraperitoneal injection of 10 mg/mL Ketamine (Abbott Laboratories) and 1 mg/mL Xylazine (Lloyd Laboratories, Shenandoah, IA, USA) in 100 µL final volume. The animals were monitored daily for a period of 4 weeks to ensure complete suppression of endogenous GnRH. After 4 weeks, all mice were sacrificed and weighed. Subsequently, prostates and testes were collected, and tissue weights were measured. The prostate was isolated together with the seminal vesicles; the bladder was removed.

Animal procedures were approved by the Ethics Committee of the University of Crete Medical School and the Veterinary Department of the Region of Crete, Greece (licence number 294320/2019) and in compliance with EU regulations on Use of Laboratory Animals.

### Hormonal treatment of men

The participants in this experiment were healthy male volunteers, recruited by advertisements in social media in September to October 2019. The inclusion criteria were 20–30 years of age, no chronic disease, drug-free, non-smoking, and BMI 20–25 kg/m^2^. The exclusion criteria were use of any narcotics for the last 3 months, previous or present use of anabolic steroids, and previous liver or heart disease or stroke. Fasting blood samples, taken before 10:00 h, were collected at the start, after 3 weeks, and after 5 weeks when a medical follow-up examination was undertaken.

Of the 48 men recruited, 33 met study criteria and were randomly assigned to either rFSH or control treatment. Background characteristics of the 33 men included in the study did not differ at baseline between the two treatment groups (Table 1). To inhibit gonadotropin secretion and action, two abdominal subcutaneous injections of degarelix (each 120 mg) were administered at the beginning of the study (Fig. 1B). Sixteen men were on the same occasion treated with 300 IU follitropin α rFSH (Gonal-f), and the rest (*n*  = 17) were untreated. Subsequently, the same dose of rFSH was self-administered three times/week for the rest of the study period, in total for 5 weeks. At 3 weeks from the start, all participants received an intramuscular injection of 1000 mg testosterone undecanoate (Nebido^®^, Bayer AG, Leverkusen, Germany) to restore testosterone levels and reduce the side effects of medical castration.

**Table 1:** Background characteristics of the study population, mean value, and range.

	Weight (kg)	Height (cm)	BMI (kg/m^2^)	Age (years)
No FSH treatment (*n* = 17)	76.3 (71.0–87.0)	182.4 (168–195)	22.7 (20.1–24.9)	23.3 (20–28)
FSH treatment (*n* = 16)	76.4 (68.4–90.5)	183.3 (175–195)	22.6 (20.5–24.9)	23.9 (20–30)

All men participated with written informed consent, that is, they were clearly informed about possible adverse effects of the treatment, for example, impaired libido, hot flushes, etc., and consented to these intermittent side effects. The study was approved by the Swedish Ethical Review Authority (2019/01942) and was also reported to www.ClinicalTrials.gov (NCT04134130).

### Clinical chemistry measurements

Biomarkers measured in human plasma/serum at the department of Clinical Chemistry, Scania University Hospital, Malmö, Sweden, included PSA, testosterone, LH, FSH, and inhibin B. The analyses were done on a Cobas 6000 instrument (Roche Diagnostics).

### RNA extraction and real-time PCR on animal tissue

Total RNA was isolated from the testis and prostate (including the seminal vesicles) tissues of the animals using TRIzol^®^ (Invitrogen) and the concentration was calculated from the absorbance at 260 and 280 nm using a NanoDrop™ 2000 spectrophotometer (Thermo Scientific™). Only samples with a 260/280-nm ratio >1.8 were processed further. The RT protocol for SYBR Green qPCR assays was followed as described in the Primescript™ RT reagent Kit (Takara Bio Inc.) to reversely transcribe 800 ng of RNA in a total reaction volume of 10 µL. Control samples lacked reverse transcriptase and RNA template, respectively. The synthesized cDNAs were diluted 1:5 with water and stored at −20°C until use. The KAPA SYBR^®^ FAST qPCR protocol (KAPA Biosystems, Wilmington, MA, USA) was followed to measure the expression levels of the genes encoding: beta-microseminoprotein (MSMB), seminal vesicle secretion 6 (Svs6), spermine-binding protein, prostate-specific homeobox gene Nkx3.1 and the PSA murine ortholog probasin (*Pbsn*). Five nanograms of DNA and reagents in a total volume of 10 μL was used in all fast ramp speed ROX-free reactions. Quantitative real-time PCR assays were carried out in a StepOnePlus™ instrument (Applied Biosystems^®^) using comparative С_T_(ΔΔС_T_) for quantitation. Ribosomal 18S gene was used as a reference. The primers (Table 2) were designed using Primer3 and BLAST tools (https://www.ncbi.nlm.nih.gov/tools/primer-blast/). Melting curve analyses that were used to monitor non-specific PCR products and data analyses, were performed using StepOne™ Software v2.3, Microsoft Office^®^ Excel andGraphPad™ Prism v7.00. The relative mRNA expression levels were calculated using the formula: 2^−ΔΔCT^ and normalized according to the normalized tissue/body weight of the sample.

**Table 2:** Primer sequences for quantitative real-time PCR.

Gene	Gene bank	Primer	Primer Sequence (‘5–3’)	Temperature (°C)	PCR product (bp)
β-microseminoprotein	NM_020597.3	Msmb F	ATCAAATGTCTGATGACTGCATGG	59.66	207
		Msmb R	TCTTCCGATCCACCACACTG	59.39	
Seminal vesicle secretory protein 6	NM_013679.2	Svs6 F	GACAGAAATGATGGAGTCACACAG	59.61	158
		Svs6 R	AGAGGATATTCTCATAACCACGGG	59.47	
PSA murine homolog probasin	NM_017471.2	Pbsn F	GTATCATGGACACGGACAACTG	59.07	228
		Pbsn R	ACATGGGAAACAATGAGTGAAAGAG	59.76	
Nkx3.1 homeobox 1	NM_010921.3	Nkx3.1 F	CCCGAGTCTGATGCACATTTTG	60.16	181
		Nkx3.1 R	CAGGGGCAGACAGGTACTTC	59.75	
Inhibin B β-chain	NM_008381.4	Inhb(β) F	CAGCTTTGCAGAGACAGATGGC	61.90	236
		Inhb(β) R	AAAGGTATGCCAGCCGCTAC	60.15	
Inhibin A	NM_010564.5	Inha F	TTTCCCAGCTACAGGTGCCA	61.42	294
		Inha R	GCCGCCAGGTGCAGTACA	62.70	

### Statistics

Comparisons between animals were made using nonparametric (Mann–Whitney test) unpaired *t*-tests with two-tailed *P* - value calculations and 95% CI using IBM SPSS Statistics 25.0 (IBM corp).

To analyze the effect of FSH on serum PSA levels in humans, PSA levels were compared between FSH-treated and untreated men at weeks 3 and 5, respectively, by analysis of covariance (ANCOVA) adjusted for baseline PSA. The effect of FSH on inhibin B levels was similarly investigated with ANCOVA adjusted for baseline Inhibin B levels. ANCOVA models were checked for constant variance and normality using residual plots with all models satisfying these assumptions.

Measurements outside the ranges of quantification were imputed as follows: FSH <0.2 IU/L adjusted to 0.1 IU/L (*n*  = 32), PSA <0.1 µg/L adjusted to 0.05 µg/L (*n*  = 5), LH <0.2 IU/L adjusted to 0.1 IU/L (*n*  = 48), testosterone <0.4 nmol/L adjusted to 0.2 nmol/L (*n*  = 2) and testosterone>51 nmol/L adjusted to 51 nmol/L (*n*  = 4). Statistical analyses were performed using R version 4.0.5 with the addition of packages ‘Hmisc’, ‘rms’, and ‘ggplot2’. *P* < 0.05 was considered a statistically significant difference.

## Results

### Hormonal treatments of animals

**Figure 2:**
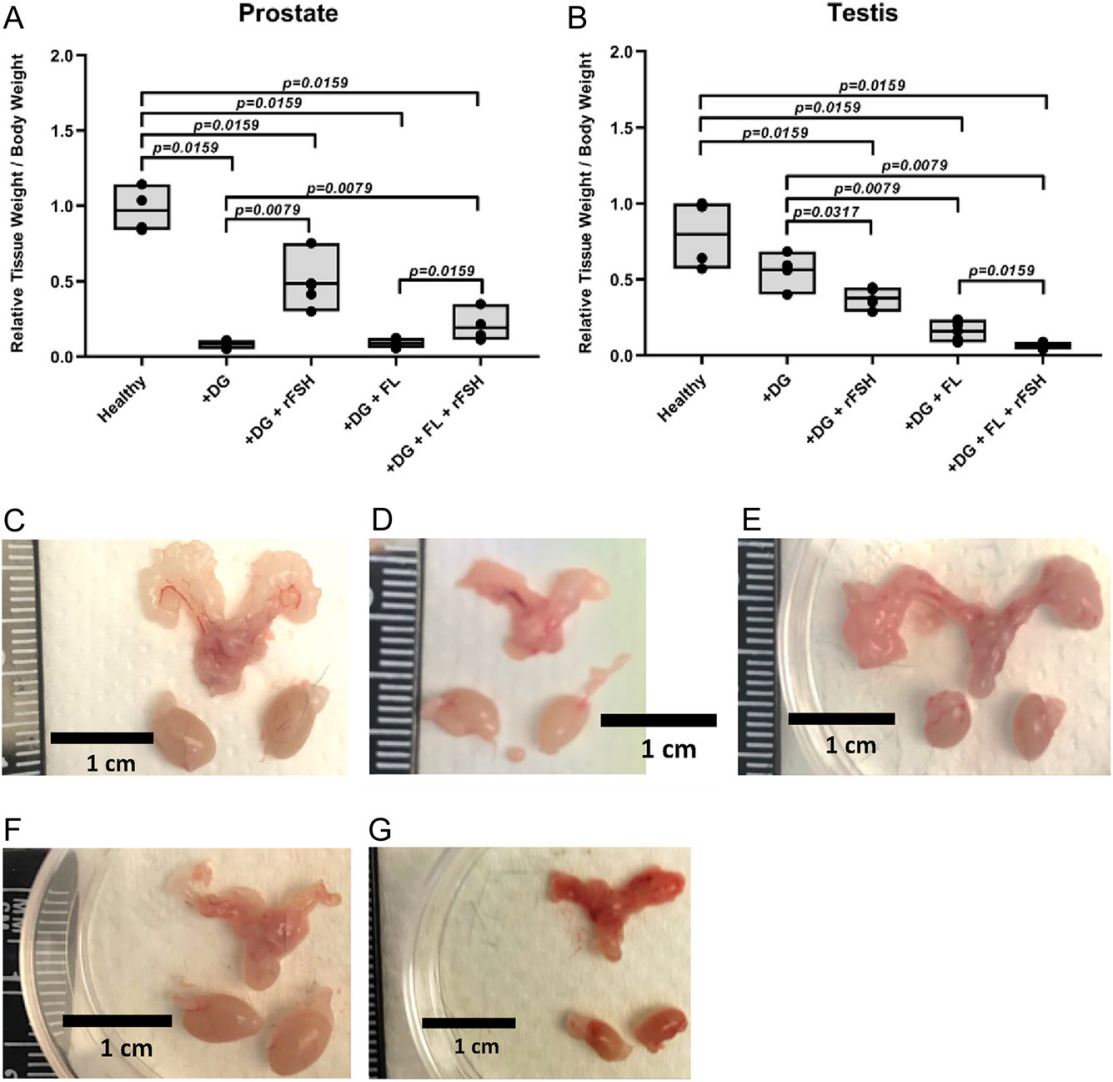
Tissue to body weight ratio of (A) prostate and (B) testis samples. Tissue samples were normalized to the mean ratio of its group divided by the mean ratio of the control group. Representative images of prostates and testes from (C) controls; (D) dgx; (E) dgx+rFSH; (F) dgx+flutamide; (G) dgx+rFSH+flutamide-treated animals.

To determine the effects of FSH on tissue homeostasis, prostate weight/body weight ratio was calculated. The ratio of each sample was first normalized to the mean ratio of the specific group divided by the mean ratio of the controls. The 91% reduction in mean prostate/body weight ratio in dgx-treated mice, compared to the controls, diminished to 50% after addition of rFSH (*P* = 0.0079; Fig. 2A). With flutamide, the 91% reduction in prostate size became 80% after addition of rFSH (*P* = 0.0159).

All chemically castrated mice also had lower testis weight/body weight ratios compared to the controls (Fig. 2B). Following the addition of rFSH, the decrease remained statistically significant and in the same range as in the mice only castrated. Representative tissue samples according to treatment modalities are shown in Fig. 2C, D, and E.

### Effect of FSH on the expression of androgen-regulated prostate genes in mice

**Figure 3:**
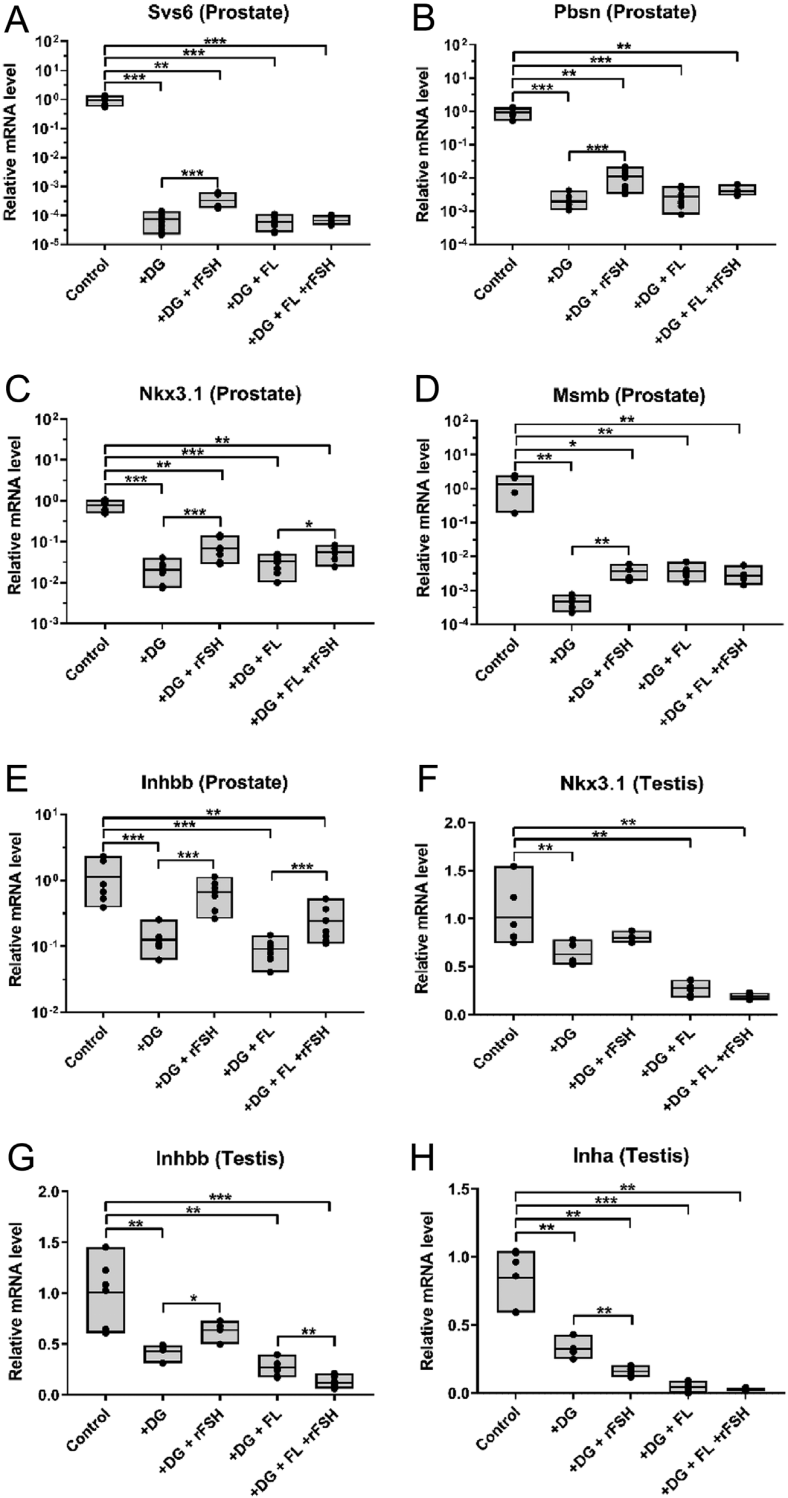
Prostatic Svs6, Pbsn, Msmb, Inhbb, and Nkx3.1 expression normalized by prostate/body weight with the mean value indicated. Testicular mRNA expression of Nkx3.1, inhibin B, and inhibin A is shown in panels (F, G, and H).

In prostate tissues of castrated mice, the expressions of the androgen-dependent genes were significantly reduced compared to controls (Fig. 3). rFSH treatment resulted in statistically significantly higher expression of the same genes in comparison to treatment without rFSH (*P* = 0.004 to *P* = 0001, depending on the gene). The addition of rFSH to treatment with dgx+ flutamide resulted in a significant increase in the expression of *Nkx3-1* and *InhbB* in the prostate compared to the corresponding treatment without rFSH.

In mouse testes, the mRNA expression levels of *Nkx3-1*, *InhbB* and *inhbA* were significantly decreased in the castrated animals compared with controls. The expression of *inhbB* in mice receiving rFSH was increased by 50% compared to those only castrated (*P* = 0.03), whereas that of *inhibin A* was not.

### Effect of FSH on PSA and inhibin B expression in men

**Figure 4:**
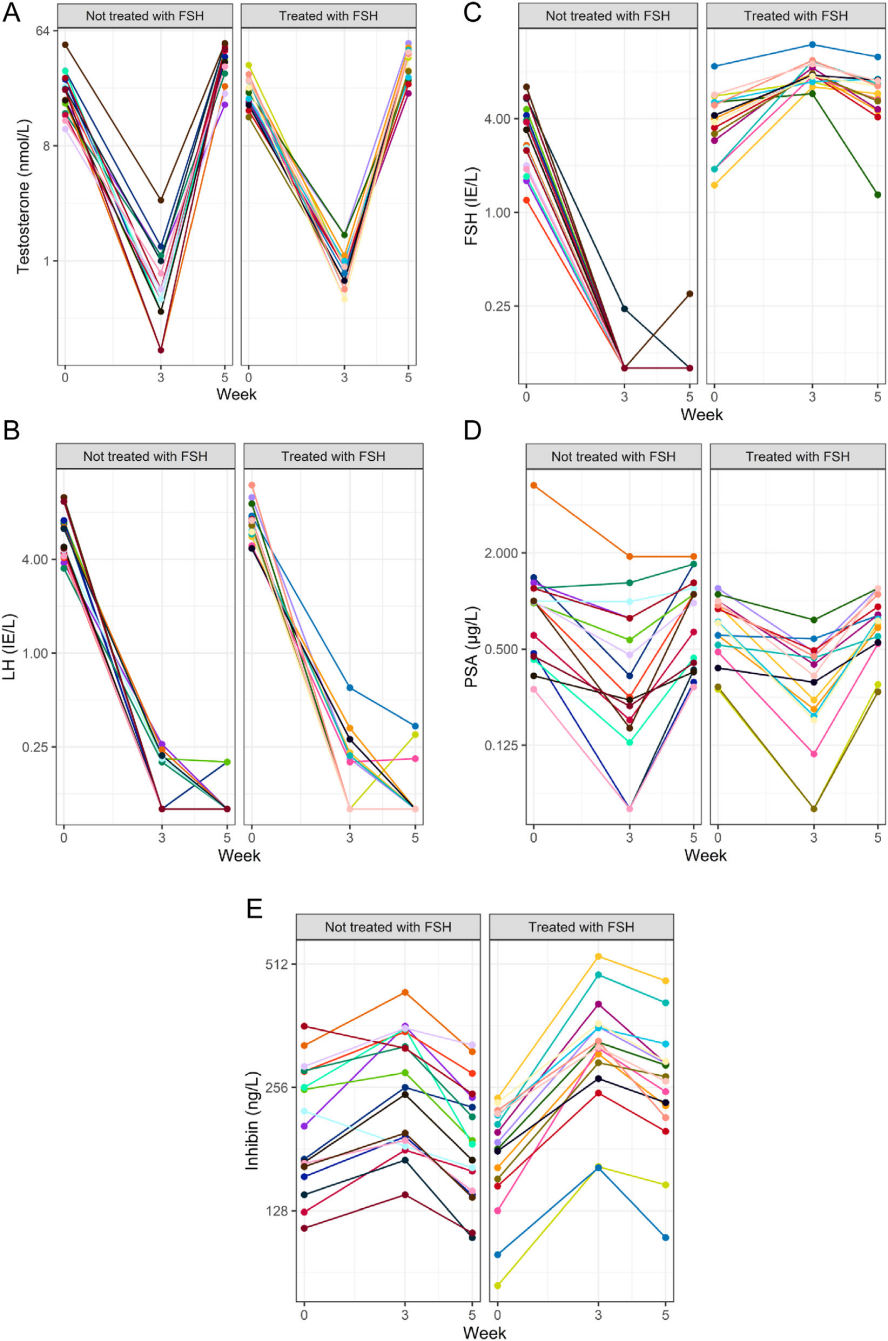
Changes in testosterone (A), LH (B), FSH (C), PSA (D), and inhibin B (E) from baseline when all men received degarelix to 3 weeks thereafter, when testosterone was administered, and after 5 weeks. Men treated with rFSH (right panel), and untreated men (left panel). Vertical axes in logarithmic scale. Plasma levels did not differ between rFSH-treated and untreated men regarding any of the hormones (*P* > 0.05), except for inhibin B (*P* < 0.0001).

After 3 weeks of antagonist treatment, all men except one had testosterone levels ≤1.6 nmol/L, turning back to baseline, or somewhat above, after testosterone supplementation (Fig. 4A and Table 3). One participant had testosterone 49.6 nmol/L at baseline, 3.0 nmol/L at week 3, and >51 nmol/L at week 5. The concentration of LH declined to almost undetectable level (<0.2 IU/L) in all men in response to dgx and remained suppressed throughout the study (Fig. 4B). In rFSH-treated men, serum FSH increased from 4.1 to 8.0 IU/L after 3 weeks, dropping to 5.9 IU/L in response to testosterone supplementation (Fig. 4C). In untreated men, FSH decreased from 3.2 IU/L in most men below the limit of detection (0.1 IU/L) in response to the antagonist and remained low throughout the study period.

**Table 3:** Concentrations of biochemical markers at baseline, after 3 weeks of antagonist treatment in the presence (*n*  = 16) and absence (*n*  = 17) of FSH, and at the end of the study when all men had received testosterone for the last 2 weeks.

Biomarker	Baseline	Week 3	Week 5
PSA (µg/L)			
FSH	0.73 (0.28–1.20)	0.33 (0.05–0.76)	0.78 (0.27–1.20)
No FSH	1.07 (0.28–5.30)	0.50 (0.05–1.90)	0.95 (0.29–1.90)
Testosterone (nmol/L)			
FSH	21.9 (13.4–34.3)	0.9 (0.5–1.6)	36.7 (20.6–51.0)
No FSH	22.2 (10.8–49.6)	0.8 (0.2–3.0)	39.5 (16.8–51.0)
LH (IU/L)			
FSH	6.9 (4.7–12)	0.2 (0.1–0.6)	0.1 (0.1–0.3)
No FSH	5.6 (3.5–10)	0.1 (0.1–0.3)	0.1 (0.1–0.2)
FSH (IU/L)			
FSH	4.1 (1.5–8.7)	8.0 (5.8–12)	5.9 (1.3–10)
No FSH	3.2 (1.2–6.4)	0.1 (0.1–0.2)	0.1 (0.1–0.3)
Inhibin B (ng/L)			
FSH	179 (84–241)	**329 (163–535)**	**260 (110–466)**
No FSH	217 (116–361)	**267 (140–437)**	**203 (110–325)**

*P* > 0.05 for all except inhibin B (*P* < 0.0001) is indicated in bold.

After 3 weeks, all men presented with a decline in PSA (Fig. 4D), which was normalized at the end of the study period in response to the testosterone substitution administered after 3 weeks. Plasma PSA levels did not differ between rFSH-treated and untreated men at week 3 (95% CI: −0.21 to 0.13 µg/L, *P* = 0.63) nor at week 5 (95% CI: −0.28 to 0.17 µg/L, *P* = 0.64). Chemical castration increased inhibin B concentrations in all men, followed by a drop after testosterone supplementation (Fig. 4E). After 3 weeks, inhibin B was 1.23-fold higher in men treated with rFSH as compared to the non-treated ones (*P* < 0.0001). Inhibin B on week 5, 2 weeks after the initiation of testosterone supplementation, was 1.28-fold higher in rFSH-treated men as compared to untreated men (*P* < 0.0001).

## Discussion

The main finding of this study was that in mice, GnRH antagonist treatment-induced reduction in prostate size was partly reversed by rFSH, leading to five times larger prostate size compared to rFSH-untreated animals. Moreover, in mice with intact adrenal androgen production, rFSH stimulated the expression of probasin, which is an orthologue to PSA. The current data support the recent report that mice, like humans, in the adrenal gland synthesize androgens able to drive androgen-dependent gene expression (8). In accordance, on top of chemical castration, additional inhibition of the residual adrenal-derived androgen action, by antiandrogen treatment, was required for complete inhibition of prostate tissue growth.

In GnRH antagonist-treated men, no functional impact of rFSH on the prostate was found, as monitored with plasma PSA levels. Whether this divergence is species related or due to other reasons, such as the duration of the antagonist treatment or the fact that adrenal androgen production was not blocked in the men, remain open questions. We could not, for ethical reasons, block adrenal steroid production in men as it might have had serious side effects. However, a previous study demonstrated that serum DHEA was strongly correlated with intra-prostatic androgen concentrations, indicating that adrenal androgens may play an important role as androgen precursors within the prostate when the testicular supply of testosterone is blocked (9).

Yet, another explanation for the negative finding could be that prostatic PSA secretion in younger healthy men is a totally testosterone-dependent and FSH-independent process. Support for this explanation comes from a similar study on GnRH antagonist-treated men, in whom a substantial decrease in PSA secretion was noted first, but later normalized when testosterone was given after three weeks, while FSH still remained suppressed (11). In a study on men assigned to either placebo or 1 month of GnRH antagonist in the absence or presence of testosterone, the main finding was similar; when the testosterone levels decreased also PSA dropped but completely recovered one month after cessation of antagonist (12). Randomization to graded doses of testosterone after GnRH agonist treatment showed no significant change in PSA at any testosterone dose, indicating that even low testosterone doses are able to maintain PSA levels (13).

Serum FSH levels were not discussed or presented in the latter study (11) and as an agonist was used, FSH was not completely suppressed. Hence, the possibility remains that FSH, in synergy with testosterone, maintained PSA production in these men. This suggestion is also in agreement with a recent finding on human prostate cancer cell line xenografts in nude mice. In antagonist suppressed mice, FSH supplementation was able to stimulate the growth of androgen-independent cell lines (PC-3, DU154) but not of the androgen-dependent cell line VCaP (14). It was concluded that the FSH effect is relatively weak in comparison to the strong androgen effect and can only be observed in the absence of the latter.

Regarding the expression of probasin and other androgen-regulated genes in mice, FSH treatment reversed the effect of the antagonist, but in animals treated with the antiandrogen flutamide, only Nkx3.1 and inhibin B responded to rFSH. These findings indicate that the FSH effects at least partly overlap with those of testosterone (8), but cannot fully substitute the androgenic effects. Stimulation of the same androgen-dependent genes by testosterone and FSH has also been demonstrated in mouse testis (6). Thus, the actions of FSH and testosterone through partly overlapping signaling pathways may be additive or synergistic. Fine-tuning of actions of the two hormones by inhibin B in the testis suggests a distinct regulation with the objective to nurture the production of mature spermatozoa. Possibly, and perhaps even likely, prostate cancer cells utilize the same process for growth and progression to an incurable disease, but in healthy men, we do not find evidence for this to be the situation.

The response of inhibin B levels to the hormonal manipulations in men deserves a comment. The data demonstrated the classical stimulatory effect of FSH on inhibin B synthesis and secretion (15). The other response was the increase of inhibin B after gonadotropin and testosterone elimination and its reversal following testosterone supplementation. The increase in inhibin B after GnRH-antagonist administration is apparently due to suppressed testosterone, which overrides the expected inhibitory effect of suppressed FSH. The role of testosterone is also supported by the reduction of inhibin B when testosterone levels were restored. Multiple studies have reported the reciprocity of testosterone and inhibin B levels in male rats and monkeys* in vivo* (16, 17), and testosterone also inhibits inhibin B production in cultured rat Sertoli cells (18). The data on humans are more variable. Inhibin B was not increased upon dgx treatment in prostate cancer patients (19) or in healthy volunteers in response to testosterone-stimulating human chorionic gonadotropin treatment (20) but the persistent decline in inhibin B in a testosterone/progestin male contraceptive trial following a rapid drop of FSH levels is in line with the animal data and our current findings (21). The mechanism of how testosterone would negatively regulate inhibin B production has not been addressed.

Although we were not able to use identical endpoints in monitoring the potential prostatic effects of FSH in the mice and men, we consider it prudent to conclude that in an experiment with a similar duration, 4–5 weeks, direct prostatic effects of FSH could only be demonstrated in mice. Whether a longer duration of treatment is needed to demonstrate FSH effects on the human prostate, or whether the human prostate is unresponsive to FSH, remains to be studied. Whether normal and malignant prostate tissue have similar response to FSH stimulation also remains to be studied. These remaining questions are important in view of the bourgeoning evidence for direct FSH actions on human prostatic cancer, as discussed above.

In summary, the current data provide novel evidence for a direct effect of rFSH on prostatic mass and gene expression in the absence of testosterone in mice. However, rFSH did not have any effect on PSA production in testosterone-suppressed young men. It remains to be explored whether these findings demonstrate a species difference in prostatic FSH action, if verification of the prostatic FSH effect in humans also requires, as in mice, complete blockage of androgen action, or whether a longer period of FSH stimulation is needed for it to have effects on the human prostate. Moreover, since the study population consisted of healthy volunteers, and not cancer patients, our results cannot be directly applied in the choice between GnRH agonist or antagonist when treating advanced prostate cancer.

## Declaration of interest

The authors declare that there is no conflict of interest that could be perceived as prejudicing the impartiality of the research reported.

## Funding

This study was supported by grants from the Swedish Cancer Society (CAN 2017/392; 2020/920), the Research Fund and Cancer Research Fund of Malmö University Hospital, ALF government grant (F2018/810), and EU Interreg NYPS 20201846. Ferring Pharmaceuticals, Saint-Prex, Switzerland supported the enrolment of human participants.
